# Performance of EMA algorithm, 2022 ACR/EULAR criteria, and EMA-ACR/EULAR algorithm in classifying pediatric ANCA-associated vasculitis: a national cohort study in China

**DOI:** 10.1007/s12519-025-00899-2

**Published:** 2025-05-10

**Authors:** Zhe Lu, Li-Wen Tan, Hong Xu, Zheng-Kun Xia, Xiao-Yun Jiang, Xiao-Chuan Wu, Fang Wang, Xiao-Rong Liu, Cheng-Guang Zhao, Xiao-Zhong Li, Jian-Hua Mao, Xiao-Wen Wang, Wen-Yan Huang, Xiao-Shan Shao, Jian-Jiang Zhang, Shi-Pin Feng, Jun Yang, Qiu Li, Ai-Hua Zhang, Mo Wang

**Affiliations:** 1 Department of Nephrology Children’s Hospital of Chongqing Medical University, National Clinical Research Center for Child Health and Disorders, Ministry of Education Key Laboratory of Child Development and Disorders, China International Science and Technology Cooperation Base of Child Development and Critical Disorders, Chongqing Key Laboratory of Pediatric Metabolism and Inflammatory Diseases, Key Laboratory of Children’s Vital Organ Development and Diseases of Chongqing Health Commission, Zhongshan 2nd Rd. 136, Chongqing, 400014 China; 2 Department of Nephrology, Children’s Hospital of Fudan University, National Pediatric Medical Center of China, Shanghai, China; 3 Department of Pediatrics, Jinling Hospital, Nanjing Medical University, Nanjing, China; 4 Department of Pediatrics, Jinling Hospital, The First School of Clinical Medicine, Southern Medical University, Nanjing, China; 5 Department of Pediatrics, Jinling Hospital, Medical School of Nanjing University, Nanjing, China; 6 Department of Pediatric Nephrology and Rheumatology, The First Affiliated Hospital of Sun Yat-Sen University, Guangzhou, China; 7 Department of Pediatrics, The Second Xiangya Hospital of Central South University, Changsha, China; 8 Department of Pediatrics, Peking University First Hospital, Beijing, China; 9 Department of Nephrology, Beijing Children’s Hospital, Capital Medical University, Beijing, China; 10 National Center for Children’s Health, Beijing, China; 11 Department of Pediatrics, Shengjing Hospital of China Medical University, Shenyang, China; 12 Department of Nephrology and Immunology, Children’s Hospital of Soochow University, Suzhou, China; 13 Department of Nephrology, Children Hospital, Zhejiang University School of Medicine, Hangzhou, China; 14 Department of Nephrology, Wuhan Children’s Hospital (Wuhan Maternal and Child Healthcare Hospital), Tongji Medical College, Huazhong University of Science & Technology, Wuhan, China; 15 Department of Nephrology and Rheumatology, Shanghai Children’s Hospital, School of Medicine, Shanghai Jiao Tong University, Shanghai, China; 16 Pediatric Nephrology Department, Guiyang Maternal & Child Health Care Hospital, Guiyang, China; 17 Department of Pediatrics, The First Affiliated Hospital of Zhengzhou University, Zhengzhou, China; 18 Department of Nephrology, Chengdu Women and Children Central Hospital, Chengdu, China; 19 Department of Rheumatology and Immunology, Shenzhen Children’s Hospital, Shenzhen, China; 20 Department of Nephrology, Children’s Hospital of Nanjing Medical University, 72 Guangzhou Road, Nanjing, 210008 China

**Keywords:** Anti-neutrophil cytoplasmic antibody-associated vasculitis, Classification criteria, Children

## Abstract

**Background:**

Anti-neutrophil cytoplasmic antibody-associated vasculitis (AAV) is a type of necrotizing vasculitis with poor prognosis, which is more severe in children. Classifying AAV patients may be helpful for diagnosis and management. However, present classification criteria for pediatric AAV are developed mainly based on adults, which have limitations in clinical practice. In this study, we introduced an updated algorithm based on the European Medicines Agency (EMA) algorithm in conjunction with the American College of Rheumatology (ACR)/European Alliance of Associations for Rheumatology (EULAR) criteria. This new approach aims to resolve the issue of duplicate classification present in the 2022 ACR/EULAR criteria and to refine the existing EMA algorithm.

**Methods:**

This study included 179 pediatric patients diagnosed with AAV across 17 centers in China. Patients were classified using the EMA algorithm, the ACR/EULAR criteria, and the EMA-ACR/EULAR algorithm. The Kappa value and Net Reclassification Index (NRI) were used to evaluate the classification performance of these criteria.

**Results:**

According to the EMA algorithm, 136 (76.0%) patients were classified with microscopic polyangiitis (MPA) and 14 (7.8%) with granulomatosis with polyangiitis (GPA), while 29 (16.2%) remained unclassifiable. According to the ACR/EULAR criteria, 145 (81.0%) patients were classified with MPA, 14 (7.8%) with GPA, 2 (1.1%) with eosinophilic granulomatosis with polyangiitis (EGPA), and 4 (2.2%) with both MPA and GPA, while 14 (7.8%) remained unclassifiable. The EMA-ACR/EULAR algorithm classified 124 patients (69.3%) as MPA, 26 (14.5%) as GPA, and 2 (1.1%) as EGPA, while 27 (15.1%) were unclassified. The Kappa values between the EMA algorithm and ACR/EULAR criteria for GPA and MPA were 0.225 [95% confidence interval (CI) 0.000–0.456, *P* = 0.003] and 0.357 (95% CI 0.196–0.518, *P* < 0.001). Compared to these two criteria, the EMA-ACR/EULAR algorithm demonstrated positive NRIs in the classification of both GPA (0.702, 95% CI 0.258–1.146, *P* = 0.002; 0.547 95% CI 0.150–0.944, *P* = 0.007) and MPA (0.425, 95% CI 0.209–0.642, *P* < 0.001; 0.519, 95% CI 0.305–0.733, *P* < 0.001).

**Conclusions:**

The EMA-ACR/EULAR algorithm addresses the limitations of the 1990 ACR criteria within the EMA framework and resolves the issue of duplicate classification in the 2022 ACR/EULAR criteria. However, further research is necessary to validate the superiority of the EMA-ACR/EULAR algorithm in the clinical classification of pediatric AAV patients.

**Graphic abstract:**

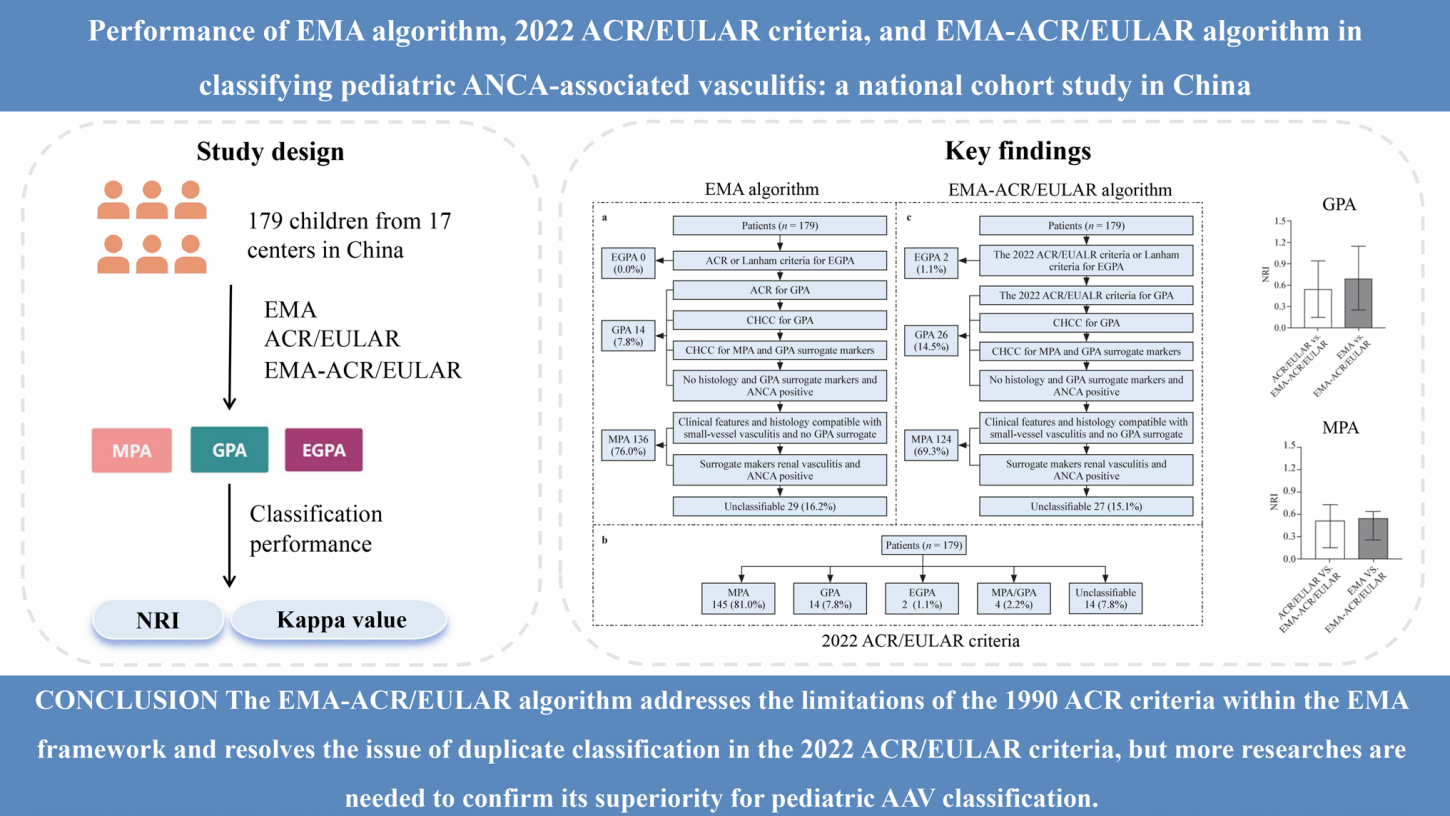

Video Abstract (MP4 19393 KB)

**Supplementary Information:**

The online version contains supplementary material available at 10.1007/s12519-025-00899-2.

## Introduction

Anti-neutrophil cytoplasmic antibody (ANCA)-associated vasculitis (AAV) is a type of necrotizing vasculitis that predominantly affects small blood vessels. Although AAV has a low incidence and is even rarer in children, it tends to manifest more severely in children than adults [1].

AAV can be classified into microscopic polyangiitis (MPA), granulomatosis with polyangiitis (GPA), and eosinophilic granulomatosis with polyangiitis (EGPA) [2]. These types exhibit differences in their pathogenesis, clinical manifestations, and treatments. Accurate identification of these subtypes is essential for effective diagnosis and management of the disease.

There are multiple classification criteria for AAV. However, most classification criteria, such as the European Medicines Agency (EMA) algorithm and the 2022 American College of Rheumatology (ACR)/European Alliance of Associations for Rheumatology (EULAR) classification criteria, are developed based on adult data and only a few studies have validated these criteria in pediatric patients. Additionally, the applicability of these classification criteria in Asian children remains uncertain due to regional and ethnic differences in AAV.

The EMA algorithm is one of the most commonly utilized classification criteria, which can maximize the classification of AAV into a single type in a sequence of steps [3]. A study reported that the EMA algorithm demonstrated high sensitivity and accuracy for pediatric GPA [4]. With the deepening understanding of AAV, the 1990 ACR criteria have become increasingly inadequate to meet current clinical needs, primarily due to their limited classification accuracy and lack of specific serum markers. Consequently, in 2022, the ACR and EULAR jointly developed new classification criteria that build upon the 1990 ACR criteria, which are intended for use in clinical research rather than for clinical management [5–7]. The 2022 ACR/EULAR criteria employ scoring systems that are more straightforward and user-friendly compared to the EMA algorithm. However, due to the absence of a specified classification sequence in the 2022 ACR/EULAR criteria, patients may be categorized into multiple subtypes simultaneously [8–10]. To resolve the issue of duplicate classification in the 2022 ACR/EULAR criteria and to enhance the EMA algorithm, we have developed the EMA-ACR/EULAR algorithm, which integrates the 2022 ACR/EULAR criteria in place of the 1990 ACR criteria within the EMA framework.

Renal involvement is common in AAV, with renal damage resulting from AAV referred to as ANCA-associated glomerulonephritis (AAGN). AAGN significantly influences the prognosis of AAV patients. About 20%–50% of pediatric patients with AAGN develop end-stage renal disease (ESRD) [11–14]. Although studies have found no significant differences in renal outcomes between MPA and GPA, the relationship between different classification criteria and renal outcomes remains unclear [12, 14].

In this study, we compared the performance of the EMA algorithm, the 2022 ACR/EULAR criteria, and the EMA-ACR/EULAR algorithm to provide some reference for the selection of classification criteria for childhood AAV. Furthermore, we investigated the correlation between these classification criteria and renal outcomes.

## Methods

### Patients

This study included 179 children diagnosed with AAV at 17 centers across China between January 1, 2012, and March 1, 2020 (Supplementary Table 1). Inclusion criteria were as follows: (1) patients meeting the 1990 ACR criteria [15, 16], the EMA algorithm [3] or the 2012 CHCC definitions [2]; and (2) children aged ≤ 18 years at the time of initial diagnosis. Patients with vasculitis secondary to systemic lupus erythematosus, drug reactions, infections, or tumors were excluded.

This study was approved by the Ethics Committee of the Children's Hospital of Chongqing Medical University (approval number: 136/2023). Because this was a retrospective study, the Ethics Committee agreed to waive informed consent.

### Data collection and definitions

Data were collected from various centers using a standardized case report form derived from an electronic medical record system. Clinical data, encompassing gender, age, clinical manifestations, laboratory tests, and renal pathology, were retrospectively gathered for all patients. Clinical manifestations included both renal and extrarenal symptoms. The laboratory tests comprised leukocyte count, eosinophil count, platelet count, blood urea nitrogen (BUN), serum creatinine (Scr), ANCA serological indicators, C-reactive protein (CRP), urine composition, and C3 levels. ANCA-testing indicators included immunofluorescence for p-ANCA or c-ANCA, and enzyme-linked immunosorbent assay (ELISA) for myeloperoxidase-ANCA (MPO-ANCA) or proteinase 3-ANCA (PR3-ANCA). Renal function was assessed using the estimated glomerular filtration rate (eGFR), calculated according to the Schwartz formula [17]. The Pediatric Vasculitis Activity Score (PVAS) was employed to evaluate initial vasculitis activity [18]. Renal pathology was characterized by glomerular, tubular, and interstitial impairment and was classified as focal, crescentic, sclerotic, or mixed by Berden's classification [19]. The study endpoint was defined as the progression to ESRD at the last follow-up. Definitions of the main clinical manifestations, renal pathology, and renal prognosis are provided in Supplementary Table 2.

### Disease classification

**Fig. 1:**
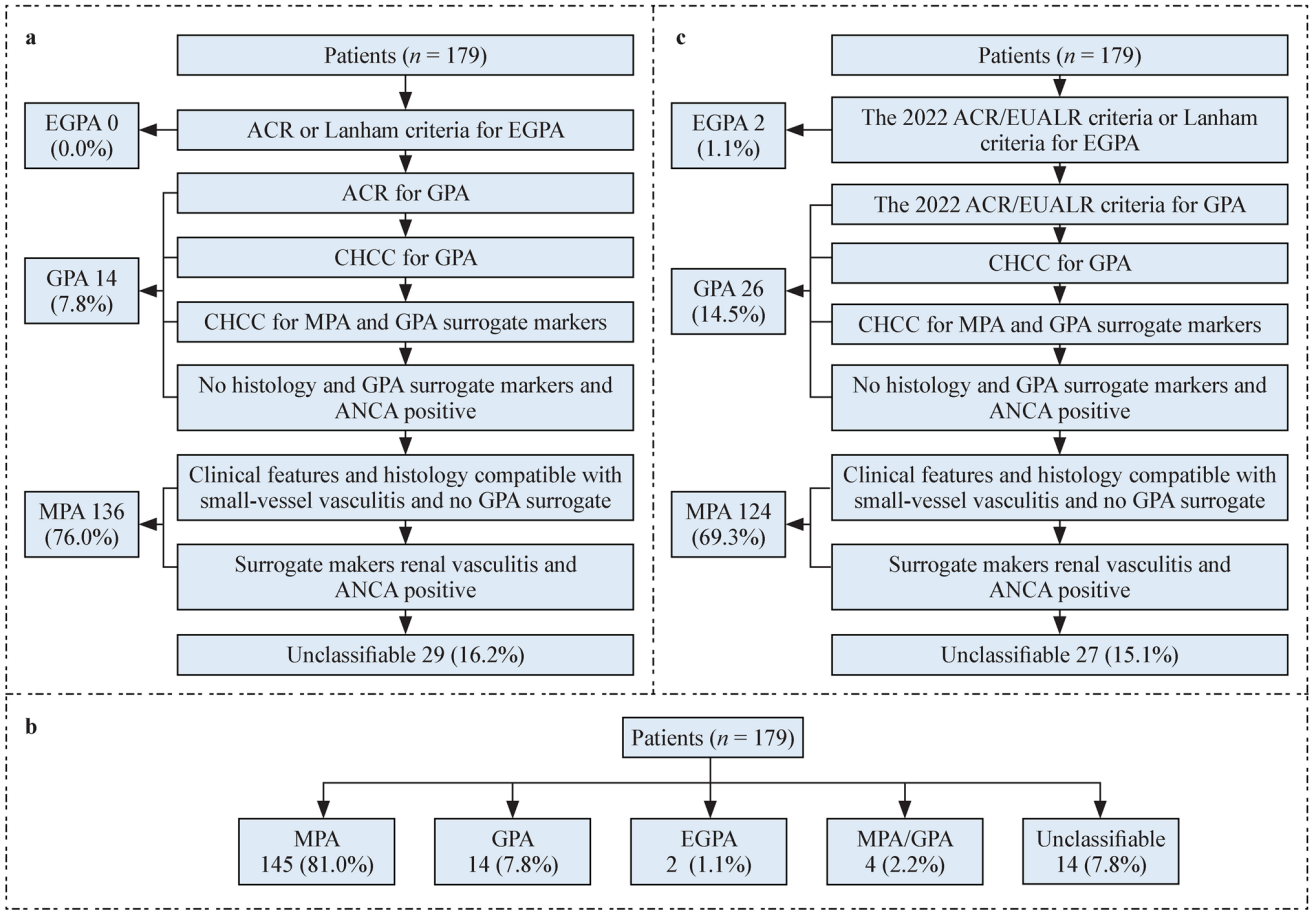
Classification according to different criteria in children with AAV. **a** Application of the EMA algorithm; **b** Application of the 2022 ACR/EULAR criteria. **c** Application of the EMA-ACR/EULAR algorithm. *EGPA* eosinophilic granulomatosis with polyangiitis, *ACR* American College of Rheumatology, *GPA* granulomatosis with polyangiitis, *CHCC* Chapel Hill Consensus Conference, *ANCA* Anti-neutrophil cytoplasmic antibody, *MPA* microscopic polyangiitis, *EULAR* European Alliance of Associations for Rheumatology

Patients were classified according to the EMA algorithm, the 2022 ACR/EULAR criteria, and the EMA-ACR/EULAR algorithm, respectively [3, 5–7]. The steps involved in the EMA-ACR/EULAR algorithm are detailed in Fig. 1. The classification was performed independently by two clinicians.

### Statistical analysis

SPSS version 26.0 (IBM Corporation, Armonk, NY, USA) was used for statistical analysis. Continuous variables were expressed as the mean ± standard deviation (SD) or median with interquartile range, and analyzed by the independent-sample t-tests and Mann–Whitney test for comparison. Categorical variables were expressed as numbers or percentages and analyzed using the Chi-squared test or Fisher's exact test. Three-year renal survival rates were estimated using the Kaplan–Meier (KM) analysis, with the log-rank test employed to compare renal survival. Two-sided *P* values of < 0.05 were considered statistically significant.

The assessment of classification criteria was performed using the Kappa consistency test and the net reclassification index (NRI). The NRI is a statistical metric used to evaluate the improvement in classification accuracy when comparing two models. In this study, the NRI was employed to evaluate the improvement of the EMA-ACR/EULAR algorithm over both the EMA algorithm and the 2022 ACR/EULAR criteria. Initially, the EMA algorithm served as the reference standard to classify all patients into MPA and Non-MPA groups. Subsequently, the ACR/EULAR criteria and the EMA-ACR/EULAR algorithm were applied to reclassify these patients, enabling the calculation of the NRIs. The NRI was computed using the following formula [20]:

$$NRI = \, \left( {n_{1} - n_{2} } \right) \, /N_{1} - \, \left( {n_{3} - n_{4} } \right) \, /N_{2}$$

Here, *N*_1_ represents patients classified as MPA by the reference standard, and* N*_2_ represents those classified as Non-MPA.* n*_1_ denoted the patients in *N*_1_ who were classified as Non-MPA by the ACR/EULAR criteria but as MPA by the EMA-ACR/EULAR criteria,* n*_2_ denoted the patients in *N*_1_ who were classified as MPA by the ACR/EULAR criteria but as Non-MPA by the EMA-ACR/EULAR criteria, *n*_3_ denoted the patients in *N*_2_ who were classified as Non-MPA by the ACR/EULAR criteria but as MPA by the EMA-ACR/EULAR criteria, and *n*_4_ denoted the patients in* N*_2_ who were classified as MPA by the ACR/EULAR criteria but as Non-MPA by the EMA-ACR/EULAR criteria.

**Fig. 2:**
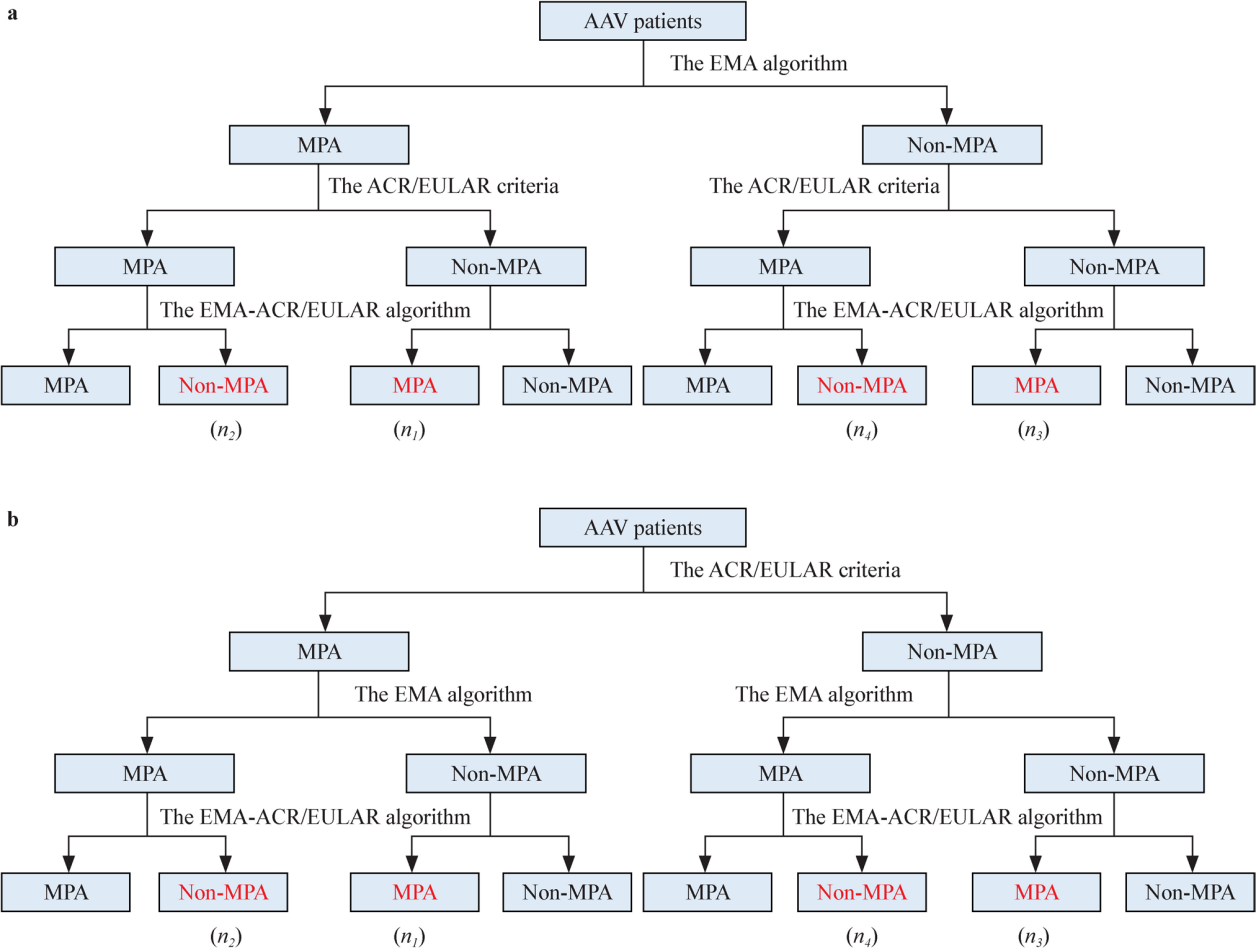
Flow chart of the NRI calculation. **a** Using the EMA algorithm as a reference standard. **b** Using the 2022 ACR/EULAR criteria as a reference standard. *AAV* Anti-neutrophil cytoplasmic antibody (ANCA)-associated vasculitis, *EMA* European Medicines Agency, *MPA* microscopic polyangiitis, *ACR/EULAR* American College of Rheumatology/European Alliance of Associations for Rheumatology

Furthermore, the ACR/EULAR criteria were also utilized as the reference standard to calculate the NRI for the EMA algorithm and the EMA-ACR/EULAR algorithm (Fig. 2). The NRIs were computed in GPA and Non-GPA patients following the same approach as described above.

## Results

### Patient characteristics

This study included 179 children diagnosed with AAV. Among these patients, 32 (17.9%) patients were male, resulting in a male-to-female ratio of 1:4.6. The median age at diagnosis was 10.1 (8.0–12.1) years. The clinicopathologic features of these patients have been described in our previous studies [21].

### Classification according to the EMA algorithm

According to the EMA algorithm, 150 patients were classified, including 136 (76.0%) with MPA and 14 (7.8%) with GPA, while 29 (16.2%) remained unclassifiable (Fig. 1a).

The characteristics of MPA and GPA as classified by the EMA algorithm are presented in Table 1. In the MPA cohort, the most frequently affected organ was the kidney (99.3%), followed by the respiratory system (45.6%) and general condition (38.2%). In the GPA cohort, the affected systems, in order of frequency, were renal (92.9%), ear-nose-throat (ENT, 85.7%), general (64.3%), and mucous membranes (57.1%). The frequency of renal involvement did not show a significant difference between the MPA and GPA groups (*P* = 0.179). However, the severity of renal injury was greater in the MPA cohort compared to the GPA cohort, as indicated by a higher proportion of patients requiring kidney replacement therapy (KRT) and a lower median eGFR at diagnosis (*P* = 0.023, 0.031). Extrarenal manifestations were observed more frequently in the GPA group, with a significantly higher occurrence of ENT symptoms compared to the MPA group (*P* < 0.001). Additionally, we observed a higher percentage of cutaneous, mucosal, and neurological involvement in the GPA cohort (*P* = 0.004, 0.006).

**Table 1:** Patient characteristics of MPA and GPA in the EMA algorithm and the 2022 ACR/EULAR criteria at baseline

Characteristics	The EMA algorithm				The 2022 ACR/EULAR criteria			
	All (*n* = 150)	MPA (*n* = 136)	GPA (*n* = 14)	*P*_1_	All (*n* = 159)	MPA (*n* = 145)	GPA (*n* = 14)	*P*_2_
Age at diagnosis (y), median (IQR)	10.87 (8.00, 12.62)	10.87 (8.00, 12.06)	10.96 (8.00, 13.00)	0.826	10.83 (8.00, 12.50)	10.42 (8.00, 12.00)	12.50 (8.00, 14.50)	0.093
Female, *n* (%)	122 (81.3)	111 (81.6)	11 (78.6)	1.000	133 (83.6)	126 (86.9)	7 (50.0)	0.001
Time between onset and diagnosis (mon), median (IQR)	1.00 (0.47, 3.27)	1.00 (0.50, 3.37)	0.90 (0.34, 2.75)	0.632	1.00 (0.40, 3.27)	1.00 (0.41, 3.85)	1.06 (0.22, 1.92)	0.556
Organ involvement, *n* (%)								
General	61 (40.7)	52 (38.2)	9 (64.3)	0.059	59 (37.1)	51 (35.2)	8 (57.1)	0.104
Renal	148 (98.7)	135 (99.3)	13 (92.9)	0.179	155 (97.5)	144 (99.3)	11 (78.6)	0.002
Edema	70 (46.7)	63 (46.3)	7 (50.0)	0.793	74 (46.5)	70 (48.3)	4 (28.6)	0.158
Proteinuria	139 (93.9)	127 (94.8)	12 (85.7)	0.204	143 (91.1)	133 (93.0)	10 (71.4)	0.027
Hematuria	144 (96.6)	131 (97.0)	13 (92.9)	0.394	144 (91.1)	135 (93.8)	9 (64.3)	0.001
Oliguria	46 (30.7)	43 (31.6)	3 (21.4)	0.629	47 (29.6)	44 (30.3)	3 (21.4)	0.695
RPGN	51 (34.2)	47 (34.8)	4 (28.6)	0.863	50 (31.6)	46 (31.9)	4 (28.6)	1.000
Patients on KRT at diagnosis	64 (43.0)	62 (45.9)	2 (14.3)	0.023	67 (42.4)	63 (43.8)	4 (28.6)	0.273
Cutaneous/Mucous membranes	34 (22.7)	26 (19.1)	8 (57.1)	0.004	38 (23.9)	34 (23.4)	4 (28.6)	0.919
Ocular	5 (3.3)	3 (2.2)	2 (14.3)	0.069	5 (3.1)	3 (2.1)	2 (14.3)	0.062
ENT	13 (8.7)	1 (0.7)	12 (85.7)	< 0.001	12 (7.5)	7 (4.8)	5 (35.7)	< 0.001
Respiratory system	67 (44.7)	62 (45.6)	5 (35.7)	0.479	68 (42.8)	62 (42.8)	6 (42.9)	0.994
Cardiovascular	4 (2.7)	3 (2.2)	1 (7.1)	0.329	4 (2.5)	4 (2.8)	0 (0.0)	1.000
Abdomen	24 (16.1)	23 (17.0)	1 (7.1)	0.564	27 (17.1)	23 (16.0)	4 (28.6)	0.410
Nervous system	16 (10.7)	11 (8.1)	5 (35.7)	0.006	16 (10.1)	15 (10.3)	1 (7.1)	1.000
Laboratory characteristics								
WBC (× 10^9^/L), median (IQR)	8.28 (6.53, 10.46)	8.19 (6.44, 10.38)	9.68 (6.97, 10.79)	0.520	8.36 (6.64, 10.76)	8.36 (6.66, 10.69)	8.25 (5.79, 12.89)	0.992
Platelet (× 10^9^/L), median (IQR)	272.00 (198.25, 377.75)	261.00 (195.75, 375.50)	286.00 (251.75, 443.25)	0.105	276.00 (199.00, 383.50)	278.00 (200.00, 382.00)	241.50 (160.50, 526.25)	0.962
Hemoglobin (g/L), median (IQR)	85.00 (65.75, 104.00)	83.50 (65.50, 103.00)	95.50 (87.00, 112.00)	0.151	86.00 (68.00, 106.00)	86.00 (69.00, 108.50)	86.50 (64.75, 108.50)	0.935
IgG (g/L), median (IQR)	9.90 (7.06, 13.50)	9.70 (6.85, 13.35)	11.40 (8.68, 14.55)	0.223	9.90 (7.05, 13.60)	9.64 (7.05, 13.30)	11.95 (9.01, 15.65)	0.156
C3 (g/L), mean ± SD	0.95 ± 0.26	0.95 ± 0.25	0.96 ± 0.35	0.893	0.95 ± 0.28	0.95 ± 0.27	0.98 ± 0.35	0.689
Albumin (g/L), mean ± SD	33.01 ± 6.28	33.01 ± 6.30	33.07 ± 6.37	0.971	33.20 ± 6.28	33.17 ± 6.46	33.54 ± 4.19	0.769
BUN (mmol/L), median (IQR)	16.1 (5.77, 30.42)	17.60 (5.85, 31.89)	7.77 (5.04, 13.19)	0.011	15.19 (5.50, 30.10)	16.10 (5.90, 30.45)	8.62 (4.24, 21.65)	0.086
Scr (μmol/L), median (IQR)	195.00 (47.70, 650.50)	264.00 (50.00, 692.00)	67.80 (41.90, 153.55)	0.020	195.00 (47.30, 653.00)	199.50 (49.22, 657.00)	65.80 (39.45, 486.35)	0.244
eGFR (mL/min/1.73m^2^), median (IQR)	29.80 (11.10, 124.10)	23.60 (10.87, 108.92)	77.20 (41.60, 181.90)	0.031	30.20 (11.10, 135.60)	26.45 (10.87, 119.07)	138.90 (24.70, 188.40)	0.018
CRP (mg/L), median (IQR)	8.40 (2.05, 26.50)	6.58 (1.95, 20.75)	15.00 (11.27, 77.30)	0.034	8.20 (2.40, 19.07)	7.77 (2.00, 18.23)	19.30 (12.00, 31.64)	0.018
*ANCA by ELISA, n* (%)								
PR3-ANCA	14 (9.3)	11 (8.1)	3 (21.4)	0.189	13 (8.2)	0 (0.0)	13 (92.9)	< 0.001
MPO-ANCA	131 (87.3)	120 (88.2)	11 (78.6)		136 (85.5)	135 (93.1)	1 (7.1)	
PR3-ANCA and MPO-ANCA	5 (3.3)	5 (3.7)	0 (0.0)		3 (1.9)	3 (2.1)	0 (0.0)	
Negative	0 (0.0)	0 (0.0)	0 (0.0)		4 (2.5)	4 (2.8)	0 (0.0)	
Missing	0 (0.0)	0 (0.0)	0 (0.0)		3 (1.9)	3 (2.1)	0 (0.0)	
ANCA by IIF, *n* (%)								
c-ANCA	7 (4.7)	6 (4.4)	1 (7.1)	0.266	8 (5.0)	1 (0.7)	7 (50.0)	< 0.001
p-ANCA	101 (67.3)	93 (68.4)	8 (57.1)		109 (68.6)	109 (75.2)	0 (0.0)	
c-ANCA and p-ANCA	3 (2.0)	2 (1.5)	1 (7.1)		2 (1.3)	1 (0.7)	1 (7.1)	
Negative	20 (13.3)	19 (14.0)	1 (7.1)		20 (12.6)	18 (12.4)	2 (14.3)	
Missing	19 (12.7)	16 (11.8)	3 (21.4)		20 (12.6)	16 (11.0)	4 (28.6)	
PVAS, median (IQR)	14.00 (12.00, 18.00)	13.00 (12.00, 18.00)	18.00 (15.50, 23.00)	0.004	13.00 (12.00, 18.00)	13.00 (12.00, 18.00)	13.50 (8.75, 19.00)	0.782
Histological class, *n* (%)								
Focal	17 (19.5)	16 (20.5)	1 (11.1)	0.811	21 (22.8)	21 (24.4)	0 (0.0)	0.551
Crescentic	28 (32.2)	24 (30.8)	4 (44.4)		37 (40.2)	33 (38.4)	4 (66.7)	
Sclerotic	18 (20.7)	17 (21.8)	1 (11.1)		18 (19.6)	17 (19.8)	1 (16.7)	
Mixed	24 (27.6)	21 (26.9)	3 (33.3)		16 (17.4)	15 (17.4)	1 (16.7)	

*P*_1_ the comparison between GPA and MPA in the EMA algorithm, *P*_2_ the comparison between GPA and MPA in the 2022 ACR/EULAR criteria. *EMA* European Medicines Agency, *ACR/EULAR* American College of Rheumatology/European Alliance of Associations for Rheumatology, *MPA* microscopic polyangiitis, *GPA* granulomatosis with polyangiitis, *IQR* interquartile range, *RPGN* rapidly progressive glomerulonephritis, *KRT* kidney replacement therapy, *ENT* ear-nose-throat, *WBC*, white blood cell, *IgG* immunoglobulin G, *BUN* blood urea nitrogen, *Scr* serum creatine, *eGFR* estimated glomerular filtration rate, *CRP* C-reactive protein, *ANCA* Anti-neutrophil cytoplasmic antibody, *IIF* indirect immunofluorescence, *ELISA* enzyme-linked immunosorbent assay, *PR3-ANCA* proteinase 3-ANCA, *MPO-ANCA* myeloperoxidase-ANCA, *PVAS* pediatric vasculitis activity score

### Classification according to the 2022 ACR/EULAR criteria

According to the 2022 ACR/EULAR criteria, 165 patients were classified, while 14 remained unclassifiable (Fig. 1b). Among the classifiable patients, there were 145 (81.0%) with MPA, 14 (7.8%) with GPA, 2 (1.1%) with EGPA, and 4 (2.2%) patients with both MPA and GPA.

Due to the small number of EGPA cases and the overlap in the classifications of four patients, we only compared the characteristics of 145 patients with MPA and 14 patients with GPA, as detailed in Table 1. The organs commonly affected in MPA, classified by the 2022 ACR/EULAR criteria, were similar to those in patients with MPA classified by the EMA algorithm. In the GPA group, renal involvement was presented in 78.6% of patients, while general (57.1%), respiratory (42.9%), and ear-nose-throat (ENT, 35.7%) involvements were also prevalent. Kidney involvement was lower in the GPA group compared to the MPA group, whereas ENT involvement was higher in the GPA group (*P* = 0.002, *P* < 0.001).

### Comparison of the EMA algorithm with the 2022 ACR/EULAR criteria

**Fig. 3:**
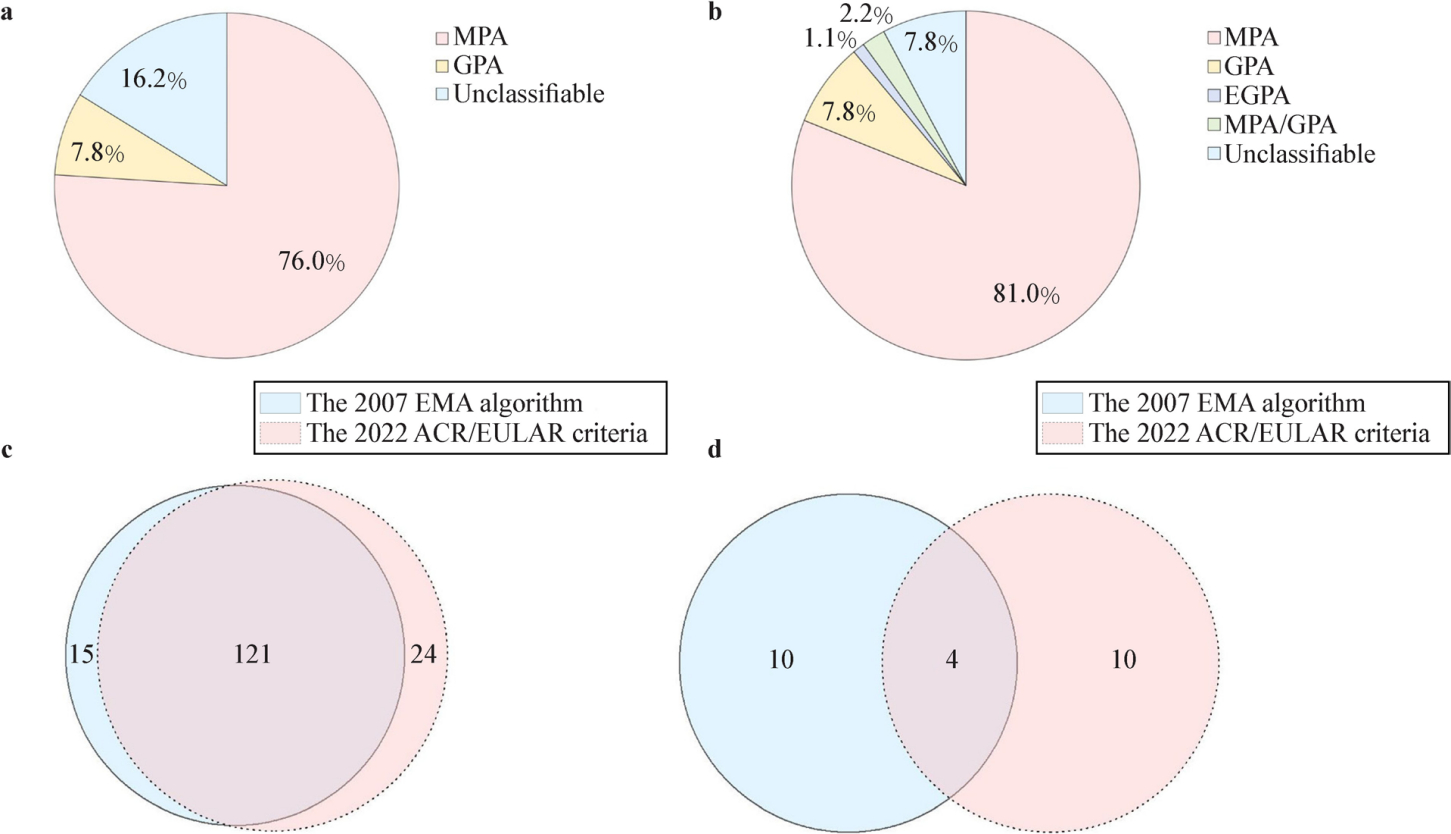
There are notable inconsistencies in classification between the EMA algorithm and the 2022 ACR/EULAR criteria. **a** Percentage of different clinical types in the EMA algorithm. **b** Percentage of different clinical types in the 2022 ACR/EULAR criteria. **c** The consistency of MPA. **d** The consistency of GPA. *MPA* microscopic polyangiitis, *GPA* granulomatosis with polyangiitis, *EGPA* eosinophilic granulomatosis with polyangiitis, *EMA* European Medicines Agency, *ACR/EULAR* American College of Rheumatology/European Alliance of Associations for Rheumatology

The number of classifiable patients according to the 2022 ACR/EULAR criteria was greater than that identified by the EMA algorithm (Fig. 3a, b). Among the 29 patients who could not be classified by the EMA algorithm, 15 (51.7%) patients were reclassified as MPA based on the 2022 ACR/EULAR criteria, which all tested positive for MPO-ANCA (or p-ANCA).

The Kappa value for GPA between the two classification criteria was 0.225 (95% CI 0.000–0.456, *P* = 0.003). Only four patients were classified as GPA using both criteria, which tested positive for PR3-ANCA (or c-ANCA) and exhibited nasal involvement (Fig. 3d). Among the patients classified as GPA according to the EMA algorithm, ten cases were not classified as GPA by the 2022 ACR/EULAR criteria. These 10 patients tested positive for MPO-ANCA (or p-ANCA) and negative for PR3-ANCA (or c-ANCA), resulting in a final total score of less than five, so they failed to meet the 2022 ACR/EULAR criteria. Nonetheless, these 10 patients could still be classified as GPA according to the EMA algorithm, as two patients met the ACR GPA classification criteria, while eight patients exhibited alternative markers for GPA and were ANCA-positive.

The Kappa value for MPA between the two classification criteria was 0.357 (95% CI 0.196–0.518, *P* < 0.001). Among the 136 patients classified as MPA by the EMA algorithm, 121 (89.0%) patients were also classified as MPA according to the 2022 ACR/EULAR criteria (Fig. 3c). However, 11 patients (8.1%) did not meet the 2022 ACR/EULAR criteria for MPA. Of these 11 patients, nine were reclassified as GPA due to positivity for PR3-ANCA (or c-ANCA) and negativity for MPO-ANCA (or p-ANCA). The remaining two patients were positive for MPO-ANCA (or p-ANCA) but had eosinophil counts ≥ 1 × 10^9^/L. Additionally, four patients (2.9%) simultaneously met the 2022 ACR/EULAR criteria for both MPA and GPA, which tested positive for MPO-ANCA (or p-ANCA) and PR3-ANCA (or c-ANCA), with renal biopsy indicating pauci-immune glomerulonephritis.

In the EMA algorithm, no patients were classified as EGPA. However, the 2022 ACR/EULAR criteria identified two cases of EGPA, both of which presented with obstructive airway disease and serum eosinophil counts ≥ 1 × 10^9^/L.

### Combination of EMA algorithm and 2022 ACR/EULAR criteria

**Fig. 4:**
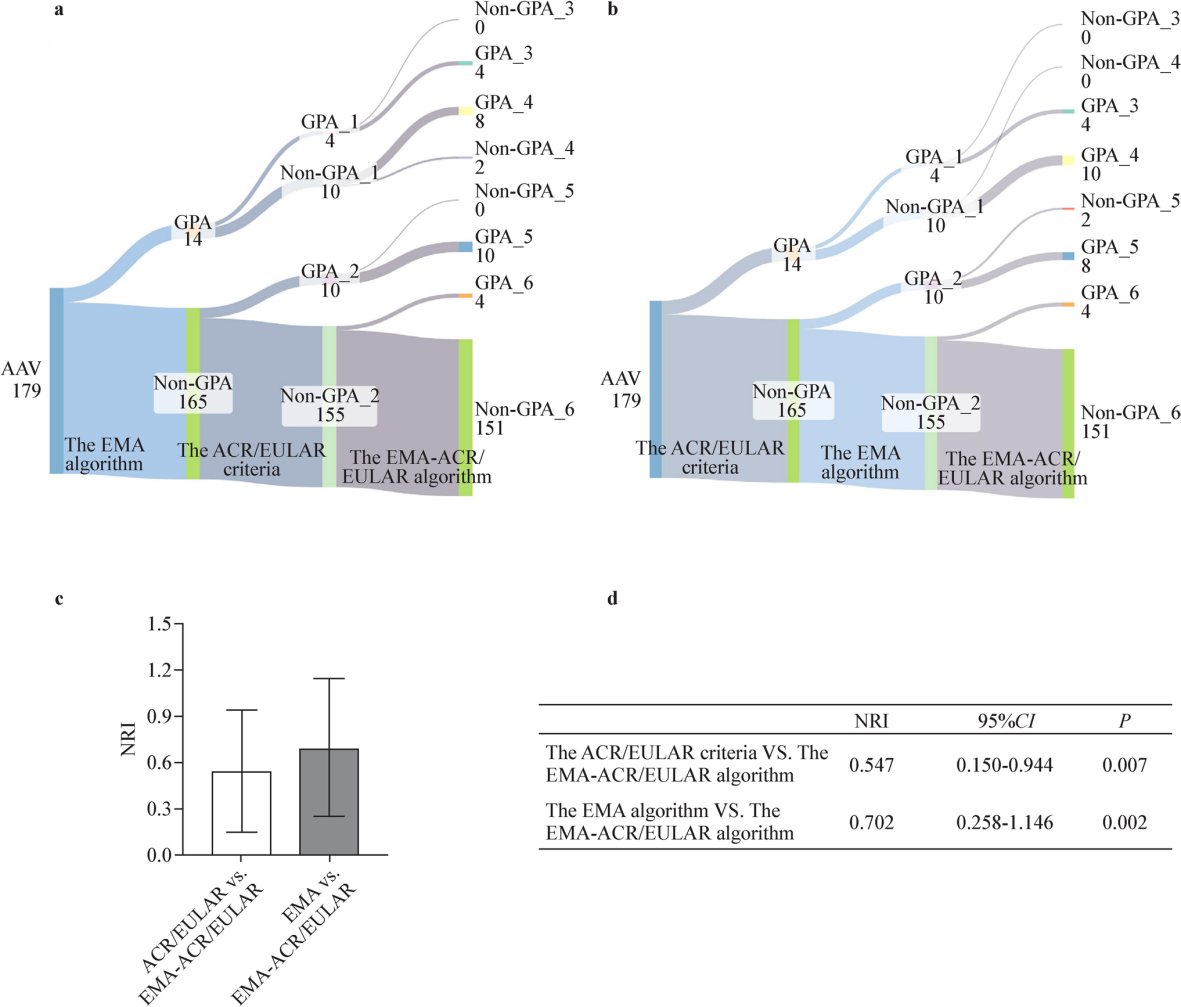
The NRIs of different classification criteria for GPA. The NRI calculation formula: *NRI* = (*GPA_4* − *Non-GPA_3*)/*GPA* − (*GPA_6* − *Non-GPA_5*)/*Non-GPA*. **a** Using the EMA algorithm as a reference standard. **b** Using the 2022 ACR/EULAR criteria as a reference standard. **c,** **d** The NRIs between the EMA-ACR/RULAR algorithm and the other two criteria. *NRI* Net Reclassification Index, *GPA* granulomatosis with polyangiitis, *EMA* European Medicines Agency, *ACR/EULAR* American College of Rheumatology/European Alliance of Associations for Rheumatology, *CI* confidence interval

**Fig. 5:**
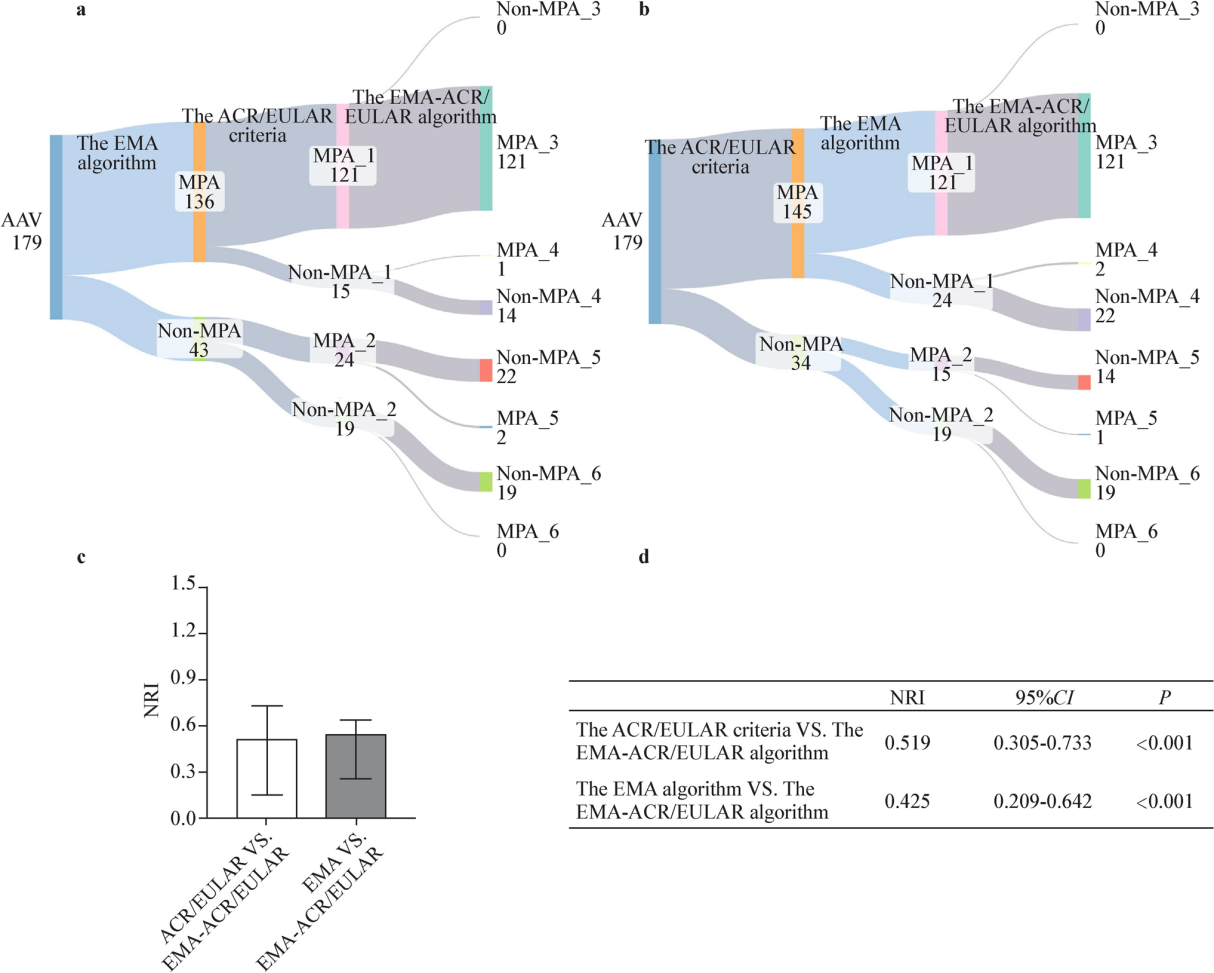
The NRIs of different classification criteria for MPA. The NRI calculation formula: *NRI* = (*MPA_4* − *Non-MPA_3*)/*MPA* − (*MPA_6* − *Non-MPA_5*)/*Non-MPA*. **a** Using the EMA algorithm as a reference standard. **b** Using the 2022 ACR/EULAR criteria as a reference standard. **c**, **d** The NRIs between the EMA-ACR/RULAR algorithm and the other two criteria. *NRI* Net Reclassification Index, *MPA* microscopic polyangiitis, *EMA* European Medicines Agency, *ACR/EULAR* American College of Rheumatology/European Alliance of Associations for Rheumatology, *CI* confidence interval

To reduce inconsistencies between the two classification criteria and address the problem of duplicate classification within the 2022 ACR/EULAR criteria, we replaced the 1990 ACR criteria in the EMA algorithm with the 2022 ACR/EULAR criteria. This new algorithm is referred to as the EMA-ACR/EULAR algorithm. The EMA-ACR/EULAR algorithm classified 124 patients (69.3%) as MPA, 26 patients (14.5%) as GPA, and 2 patients (1.1%) as EGPA, while 27 patients (15.1%) were unclassified (Fig. 1c). The NRIs for the EMA-ACR/EULAR algorithm, compared to the EMA algorithm, were 0.702 (95% CI 0.258–1.146, *P* = 0.002) for GPA and 0.425 (95% CI 0.209–0.642, *P* < 0.001) for MPA. Similarly, the NRIs for the EMA-ACR/EULAR algorithm, relative to the 2022 ACR/EULAR criteria, were 0.547 (95% CI 0.150–0.944, *P* = 0.007) for GPA and 0.519 (95% CI 0.305–0.733, *P* < 0.001) for MPA (Figs. 4, 5).

### Comparison of renal outcomes between the three classification criteria

**Fig. 6:**
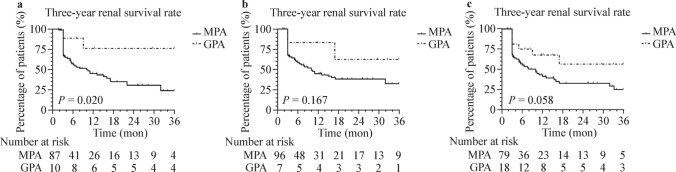
MPA had a lower three-year renal survival rate compared to GPA in the EMA algorithm. **a** KM curve of the time to ESRD in MPA and GPA according to the EMA algorithm. **b** KM curve of the time to ESRD in MPA and GPA according to the 2022 ACR/EULAR criteria. **c** KM curve of the time to ESRD in MPA and GPA according to the EMA-ACR/EULAR algorithm. *MPA* microscopic polyangiitis, *GPA* granulomatosis with polyangiitis

To understand the relationship between these classification criteria and renal prognosis, we compared the renal survival of MPA and GPA based on different criteria. In the EMA algorithm, the 2022 ACR/EULAR criteria, and the EMA-ACR/EULAR algorithm, 60 (55.5%), 63 (55.6%), and 59 (54.6%) patients developed ESRD, with median follow-up times of 25.10, 22.07, and 25.50 months, respectively. The difference in three-year renal survival between MPA and GPA in the EMA algorithm was statistically significant (*P* = 0.020) (Fig. 6a). In contrast, the difference in three-year renal survival between MPA and GPA in the 2022 ACR/EULAR criteria and the EMA-ACR/EULAR algorithm was not statistically significant (*P* = 0.167 and *P* = 0.058, respectively) (Fig. 6b, c).

## Discussion

AAV is an autoimmune vasculitis with a poor prognosis and a tendency to progress more rapidly in children. Accurate classification of pediatric AAV patients is essential for purposeful monitoring of organ damage and improving patient outcomes. In this study, 179 pediatric patients with AAV from 17 centers across China were included and classified using the EMA algorithm, ACR/EULAR criteria, and the EMA-ACR/EULAR algorithm to explore appropriate classification criteria for children.

Our findings indicated that MPA was the most prevalent clinical subtype, aligning with prior research in Asian [13, 22–24]. The kidney was the most frequently affected organ, and renal impairment was found to be more severe in cases of MPA than GPA. Moreover, ENT symptoms were more common in patients with GPA than those with MPA. Considering that distinct clinical types of AAV are associated with varying risks of organ damage, clinicians should tailor their focus to specific types of organ damage based on clinical classification. It is helpful to improve the prognosis by effectively differentiating these types and implementing targeted observation and treatment.

Although the EULAR-Pediatric Rheumatology European Society (PRES)-Pediatric Rheumatology International Trials Organization (PRINTO) criteria for childhood GPA was proposed in 2008, this system did not establish criteria for MPA and EGPA, which limits its applicability, particularly in Asian regions where MPA is predominant [25]. Therefore, our aim was to explore suitable classification criteria for pediatric patients based on the existing classification criteria for adults. We utilized the EMA algorithm and the 2022 ACR/EULAR criteria to classify patients and compared the classification results derived from both criteria. Among the patients not classified by the EMA algorithm, more than half could be reclassified as MPA using the 2022 ACR/EULAR criteria.

In addition, we found that when using the EMA algorithm and the 2022 ACR/EULAR criteria for classification, there were differences in the population that could be classified into the same type by the two classification methods, especially in the classification of GPA patients. Among the patients classified as GPA by the EMA algorithm, 71.4% were not classified as GPA under the 2022 ACR/EULAR criteria due to negative results for PR3-ANCA (or c-ANCA). In contrast, the EMA algorithm does not restrict ANCA types, allowing more patients with at least one alternative marker as well as a positive ANCA result, to be classified as GPA. When applying the 2022 ACR/EULAR criteria for MPA, 11.0% of patients classified as MPA under the EMA algorithm were not classified as MPA. The majority of these patients tested negative for MPO-ANCA (or p-ANCA). We infer that the differences in classification between the criteria may be due in part to the emphasis placed by the 2022 ACR/EULAR criteria on ANCA specificity (PR3 vs. MPO), which distinguishes MPA from GPA.

Some researchers have argued that the 2022 ACR/EULAR criteria assign an excessively high score for ANCA positivity and trivial the typical histopathological features and clinical manifestations of GPA [10, 26]. Furthermore, studies conducted in South Korea and Japan revealed that the consistency between the 2022 ACR/EULAR criteria and previous classification criteria for GPA was lower than expected [8, 26]. In Asia, MPO-ANCA positivity is common, and an overemphasis on ANCA types may lead to the oversight of MPO-positive GPA cases.

In our study, some patients were classified as MPA and GPA simultaneously because they met the MPA and GPA scores in the 2022 ACR/EULAR criteria, a finding also reported in the adult cohort [8, 10, 26]. Duplicate classification is, therefore, a challenge that needs to be addressed by the 2022 ACR/EULAR criteria. To solve this problem, we used a combination of the 2022 ACR/EULAR criteria and the EMA algorithm.

Our analysis of data from the reclassification of the Chinese AAV cohort shows two advantages of the EMA-ACR/EULAR algorithm. On the one hand, within the EMA framework, the 1990 ACR criteria have been superseded by the 2022 ACR/EULAR criteria, addressing the limitations to 1990 ACR criteria while enhancing classification accuracy. On the other hand, the EMA-ACR/EULAR algorithm solves the problem of duplicate classification in the 2022 ACR/EULAR criteria. These two advantages have been shown by NRI's statistical analysis of the improvement of the old and new criteria [20]. The NRIs of the EMA-ACR/EULAR algorithm were both positive compared with the EMA algorithm and the 2022 ACR/EULAR criteria, which exactly reflects the advantages of the EMA-ACR/EULAR algorithm. However, there was still a small number of children who cannot be classified by the EMA-ACR/EULAR algorithm, which indicates that children with unique or mild symptoms are still in the pre-diagnosis state based on adult diagnostic criteria, so these criteria may not be fully applicable to pediatric patients. Future work should focus on developing criteria for classifying early symptoms in pediatric patients.

The severity of renal injury is widely recognized as a critical factor affecting the prognosis of patients with AAV. However, whether there are differences in renal outcomes among different clinical types remains controversial [12, 14]. This study investigated the association between clinical types and renal outcomes in pediatric patients classified with different classification criteria. Our findings indicate a significant difference in renal survival between MPA and GPA only when applying the EMA algorithm. Although the EMA-ACR/EULAR algorithm is not superior in predicting renal prognosis, the significance of AAV classification criteria lies in clinical classification rather than severity classification, so the classification criteria are not necessarily related to prognosis. To predict the prognosis of AAV patients, it is also necessary to comprehensively consider clinical manifestations, pathological features, and biological indicators to build a more accurate risk prediction model. Our team has constructed predictive models for renal outcomes in AAGN in previous studies [21].

This study has several limitations. First, its retrospective design resulted in some patients being uncategorized or misclassified due to missing imaging and histopathological information. Second, the differences in the regional distribution of AAV reduced the sample size of GPA cases in this study, which may introduce bias in the results. Consequently, the conclusions drawn from this study should be validated through larger sample sizes and extended follow-up periods.

In conclusion, we used the combined use of the 2022 ACR/EULAR criteria and EMA algorithm to conduct a classification study in the children's AAV cohort. NRI analysis showed that EMA-ACR/EULAR algorithm not only effectively solved the limitations of the 1990 ACR criteria within the framework of EMA, but also improved the duplicate classification of the 2022 ACR/EULAR criteria, reflecting the advantages of the EMA-ACR/EULAR algorithm. However, whether the clinical classification of EMA-ACR/EULAR algorithm in pediatric AAV patients has advantages needs to be further verified by more studies in children. In the future, it is necessary to establish classification criteria that meet the early symptoms of pediatric patients and achieve early diagnosis and treatment to ultimately improve the prognosis of pediatric AAV.

## Supplementary Information

Below is the link to the electronic supplementary material.

Supplementary file1 (DOCX 15 KB)

## Data Availability

The data underlying this article cannot be shared publicly due to privacy and ethical restrictions. The data will be shared on reasonable request to the corresponding author.
