# Supplementary material for: Performance of EMA algorithm, 2022 ACR/EULAR criteria, and EMA-ACR/EULAR algorithm in classifying pediatric ANCA-associated vasculitis: a national cohort study in China
Source: World J Pediatr. 2025 May 10;21(4):372–85. doi: 10.1007/s12519-025-00899-2 (PMC12103362; doi:10.1007/s12519-025-00899-2)
Supplement: Supplementary file 2 — Supplementary file1 (DOCX 15 KB) [file 12519_2025_899_MOESM1_ESM.docx]

Supplementary Table 1 Number of cases in each center.

| Centers | Number of cases |
| --- | --- |
| Department of Nephrology, Children's Hospital of Fudan University | 25 |
| Department of Pediatrics, Jinling Hospital, Nanjing Medical University | 24 |
| Department of Pediatric Nephrology and Rheumatology, the First Affiliated Hospital of Sun Yat-sen University | 22 |
| Department of Nephrology, Children's Hospital of Chongqing Medical University | 21 |
| Department of Pediatrics, the Second Xiangya Hospital of Central South University | 16 |
| Department of Pediatrics, Peking University First Hospital | 12 |
| Department of Nephrology, Children's Hospital of Nanjing Medical University | 11 |
| Department of Nephrology, Beijing Children's Hospital | 10 |
| Department of Pediatrics, Shengjing Hospital of China Medical University | 7 |
| Department of Nephrology and Immunology, Children's Hospital of Soochow University | 7 |
| Department of Nephrology, the Children Hospital of Zhejiang University School of Medicine | 6 |
| Department of Nephrology, Wuhan Children's Hospital | 6 |
| Department of Nephrology and Rheumatology, Shanghai Children's Hospital | 5 |
| Department of Nephrology and Immunology, Guiyang Maternal & Child Health Care Hospital | 4 |
| Department of Pediatrics, the First Affiliated Hospital Pediatrics of Zhengzhou University | 3 |
| Department of Pediatric Nephrology, Chengdu Women's and Children's Central Hospital | 2 |
| Department of Rheumatology and Immunology, Shenzhen Children's Hospital | 2 |

Supplementary Table 2 The definitions of the main clinical manifestations, renal pathology and renal prognosis.

| Item | Definition |
| --- | --- |
| Manifestation |  |
| Proteinuria | ≥0.15 g/24 hours, urine protein/Cr ≥2.0 mg/ mg, or positive in urinalysis |
| Hematuria | >3 cells/highpower field, >17 cells/μL on urinalysis, or red cell casts |
| Oliguria | daily urination volume of school-age children is <400mL/m^2^, preschool children is <300mL/m^2^ |
| RPGN | the eGFR decline of more than 50% in less than three months |
| Renal pathology |  |
| Normal glomerulus | no scarring, crescents, or fibrinoid necrosis within the glomeruli |
| Cellular crescent | purely cellular lesions or with >10% cellular components |
| Fibrous crescent | >90% of a crescent consists of extracellular matrix |
| Segmental glomerulosclerosis | <50% of the glomerulus sclerosed |
| Global glomerulosclerosis | >50% of the glomerulus sclerosed |
| Renal prognosis |  |
| ESRD | the eGFR <15 ml/(min-1.73m^2^) or renal replacement therapy lasting more than 3 months |

RPGN rapidly progressive glomerulonephritis, eGFR estimated glomerular filtration rate, ESRD end-stage renal disease.
